# Circulating Toll-like receptor and cytokine profiles and genetic polymorphisms in a Chinese sepsis cohort: potential links to neuroinflammation and blood–brain barrier dysfunction

**DOI:** 10.3389/fimmu.2026.1762637

**Published:** 2026-03-02

**Authors:** Wu Fan, Rongfen Zhao, Jiaye Liu, Ruixiang Luo, Yun Chen

**Affiliations:** 1 Department of Clinical Laboratory, Suzhou New District People’s Hospital, Suzhou, Jiangsu, China; 2 Huangdai Town Community Health Service Center, Suzhou, Jiangsu, China

**Keywords:** blood–brain barrier dysfunction, cytokines, genetic polymorphism, neuroinflammation, PCR–RFLP, sepsis, Toll-Like receptors

## Abstract

**Background:**

Sepsis is characterised by a dysregulated host response to infection and remains a major cause of morbidity and mortality. Toll-like receptor (TLR)–mediated inflammatory signalling can amplify systemic cytokine release and has been implicated in sepsis-associated brain dysfunction through neuroinflammation and blood–brain barrier (BBB) impairment. This study examined circulating TLR/cytokine profiles and common genetic polymorphisms in key inflammatory genes in patients with sepsis.

**Methods:**

In this hospital-based case–control study, 480 adult patients with sepsis admitted to the intensive care unit and 840 age- and sex-matched healthy controls were enrolled. Serum levels of TLR2, TLR4, TLR9, IL-1β, IL-6, IL-8, IL-10, TNF-α and IFN-γ were quantified by enzyme-linked immunosorbent assay at the time of sepsis diagnosis. Genotypes of candidate polymorphisms in TLR and cytokine-related genes were determined using a classical polymerase chain reaction–restriction fragment length polymorphism (PCR–RFLP) approach. Hardy–Weinberg equilibrium was evaluated in the control group. Associations between polymorphisms and sepsis susceptibility were assessed using multivariable logistic regression adjusted for age and sex, and p values were interpreted with consideration of multiple comparisons, including conservative Bonferroni correction in sensitivity analyses.

**Results:**

Compared with controls, patients with sepsis exhibited significantly higher serum levels of TLR2, TLR4, TLR9, IL-1β, IL-6, IL-8, IL-10 and TNF-α (all p < 0.05), whereas IFN-γ levels were not significantly different. Several polymorphisms were associated with increased sepsis risk, including TLR2 −196 to −174 del, TLR4 rs1927911, TLR9 rs352140, TLR9 rs574836, IL-1B +3954 C/T, IL-6 −174 G/C, IL-10 −1082 G/A, IL-10 −819 T/C, TNF-α −308 G/A and IFN-γ +874 A/T (all p < 0.05), and a subset of these associations remained statistically significant after conservative correction for multiple testing.

**Conclusion:**

Circulating up-regulation of the TLR–cytokine axis and susceptibility-associated polymorphisms in TLR and cytokine genes support a genetic–inflammatory framework for sepsis in this Chinese cohort, with potential relevance to pathways implicated in sepsis-related neuroinflammation and BBB dysfunction. Because central nervous system involvement and BBB integrity were not directly measured in this study, these links should be regarded as inferential. The present findings motivate further studies incorporating direct central nervous system and BBB endpoints and external validation cohorts, and may inform future biomarker-guided risk stratification strategies.

## Introduction

Sepsis is a life-threatening syndrome that arises from a dysregulated host response to infection and can rapidly progress to organ dysfunction and septic shock (1). Profound disturbances in macrocirculation and microcirculation, together with metabolic derangements, contribute to impaired tissue perfusion and multisystem injury in severe cases. Despite advances in early recognition and standardised management, septic shock remains associated with substantial mortality, highlighting the need for more informative biomarkers and mechanistic indicators to support risk stratification and severity assessment (2). In addition to peripheral organ failure, sepsis can involve the central nervous system, where systemic inflammation is thought to precipitate neuroinflammation and compromise blood–brain barrier (BBB) integrity, thereby contributing to sepsis-associated brain dysfunction. However, the inflammatory and genetic determinants that shape these immune-driven processes in clinical sepsis are still not fully characterised, and many human studies rely on systemic markers rather than direct measurements of central nervous system (CNS) or BBB alterations.

Current concepts of sepsis immunopathology emphasise a dynamic and heterogeneous immune trajectory. Pro-inflammatory and anti-inflammatory programmes often overlap in time rather than occurring as strictly sequential phases, and the balance between hyperinflammation and immunosuppression may shift early during the disease course (3, 4). Circulating cytokines and related inflammatory mediators are therefore not simply byproducts, but active components of sepsis pathogenesis, and their levels have been associated with disease severity and prognosis in multiple settings (5). Nevertheless, many clinical studies have focused on a limited set of candidate cytokines or single pathways. This leaves gaps in the integrated characterisation of key inflammatory mediators and in understanding how these mediators, together with host factors, may contribute to the systemic milieu that favours neuroinflammation and BBB dysfunction in sepsis (6–10). Toll-like receptors (TLRs), as major pattern-recognition receptors, initiate innate immune signalling and regulate downstream cytokine production through canonical pathways such as MyD88- and NF-κB–dependent cascades, which can influence endothelial activation, microvascular injury and BBB disruption during systemic infection (11–14).

Genetic variation in TLR and cytokine-related genes represents an additional layer of heterogeneity in the host response to infection. Single-nucleotide polymorphisms (SNPs) in TLR2, TLR4, TLR9, IL-1B, IL-6, IL-10, TNF-α and IFN-γ have been examined in relation to sepsis susceptibility or outcome in different cohorts, with several reports suggesting that specific alleles may modify receptor expression, cytokine production or downstream signalling (6–14). However, many published studies have evaluated only a small number of polymorphisms in relatively limited samples, often without parallel measurement of circulating TLR and cytokine profiles, and data from larger and ethnically diverse populations remain limited. In particular, comprehensive analyses integrating TLR expression, cytokine levels and common polymorphisms in patients with sepsis from Chinese populations are still relatively scarce, even though genetic background, environmental exposures and infection patterns may differ from those in previously studied cohorts.

Building on this rationale, the present hospital-based case–control study investigated circulating expression patterns of TLR2, TLR4, TLR9 and a panel of inflammatory cytokines in adult patients with sepsis and healthy controls, and further evaluated whether common polymorphisms in TLR- and cytokine-related genes are associated with susceptibility to sepsis in a Chinese population. By linking systemic TLR–cytokine profiles with host genetic variation, we sought to provide a more integrated view of inflammatory and genetic determinants in sepsis, and to explore their possible relevance to pathways proposed for sepsis-related neuroinflammation and BBB dysfunction, while recognising that CNS involvement and BBB integrity were not directly assessed in this study.

## Materials and methods

### Study design, setting, and participants

This hospital-based case–control study enrolled adult patients with sepsis who were admitted to the intensive care unit (ICU) of People’s Hospital of Suzhou New District (Suzhou, Jiangsu, China) between January 2020 and May 2025. A total of 480 patients meeting Sepsis-3 diagnostic criteria were included in the sepsis group. The control group comprised 840 age- and sex-matched apparently healthy individuals recruited from the hospital physical examination centre during the same period.

All patients with sepsis received standardised care according to the Chinese Guidelines for the Emergency Treatment of Sepsis/Septic Shock (2018), including early empiric broad-spectrum antibiotics and initial fluid resuscitation. The study protocol was approved by the Medical Ethics Committee of People’s Hospital of Suzhou New District, and written informed consent was obtained from all participants or their legal guardians before blood collection.

### Case definition and eligibility criteria

Sepsis was defined according to the Sepsis-3 consensus definition (15). Patients with a confirmed or suspected infection and a Sequential Organ Failure Assessment (SOFA) score ≥ 2 at presentation were eligible. Patients were included if they (i) met Sepsis-3 criteria, (ii) were admitted to the ICU for the first time during the index hospitalisation, and (iii) had a symptom duration ≤ 24 h prior to ICU admission.

Exclusion criteria were: (i) age < 18 years or > 90 years; (ii) symptom duration > 24 h before ICU admission; (iii) death, discharge or transfer within 24 h after admission; or (iv) withdrawal or limitation of treatment precluding completion of sampling and data collection.

Healthy controls had no evidence of acute infection at recruitment and were free of severe systemic disease based on routine clinical examination, laboratory tests and medical history.

### Clinical data collection and potential confounders

Demographic characteristics (age, sex, body mass index [BMI]) and clinical histories (including diabetes, hypertension, smoking and alcohol use) were recorded at enrolment. For patients with sepsis, clinical severity and infection-related characteristics were collected at diagnosis, including SOFA score (as an index of organ dysfunction), presumed infection source/site and microbiological results when available (pathogen category based on routine cultures).

These variables were prespecified as potential confounders because they may influence cytokine expression and/or genetic association signals. However, detailed information on infection source/site, pathogen category and SOFA score was not available for all participants. Therefore, these covariates were summarised descriptively and considered when interpreting the findings, but were not incorporated into the primary regression models. Their potential confounding effects are explicitly discussed as part of the study limitations.

### Blood sampling, processing and storage

For patients with sepsis, peripheral venous blood (3–5 mL) was collected at the time of sepsis diagnosis (i.e. at enrolment) into serum separation tubes. Samples were allowed to clot at room temperature and then centrifuged to isolate serum. The serum supernatant was transferred into sterile 1.5 mL microtubes using aseptic technique, labelled with anonymised study IDs and stored at −80°C until analysis.

The same sampling and processing workflow was applied to healthy controls. To minimise pre-analytical variability, samples were thawed on ice and mixed gently before assays, and repeated freeze–thaw cycles were avoided.

### Serum measurements by ELISA

Serum concentrations of TLR2, TLR4, TLR9, IL-1β, IL-6, IL-8, IL-10, TNF-α and IFN-γ were measured using double-antibody sandwich enzyme-linked immunosorbent assays (ELISAs) according to the manufacturers’ instructions. Briefly, reagents and samples were equilibrated to room temperature, and standard solutions were prepared to generate calibration curves. Serum samples were added to pre-coated microplates (with dilution when required by the assay range). After incubation, plates were washed to remove unbound material, followed by addition of detection antibody and streptavidin–HRP conjugate.

Colour development was achieved by substrate reaction and terminated with stop solution. Optical density was read using a microplate reader at the recommended wavelength, and concentrations were calculated from the standard curves. Standards and samples were measured in duplicate, and assay acceptance followed prespecified quality criteria, including adequate standard-curve fit and consistency between duplicates.

### DNA extraction and SNP selection

Genomic DNA was extracted from peripheral blood using a commercial kit suitable for PCR-based genotyping, following the manufacturer’s instructions. DNA concentration and purity were assessed spectrophotometrically, and samples not meeting quality thresholds were re-extracted.

Candidate polymorphisms in TLR- and cytokine-related genes were selected *a priori* based on biological plausibility and prior literature in inflammation- and infection-related phenotypes. The panel included variants within TLR2, TLR4, TLR9, IL-1B, IL-6, IL-10, TNF-α and IFN-γ loci that have been reported or hypothesised to influence receptor expression, cytokine production or downstream signalling (6–14). Detailed primer sequences, restriction enzymes, annealing temperatures and expected fragment sizes for each locus are provided in **Supplementary Table S1**.

### Genotyping by PCR–RFLP

Genotypes were determined using classical polymerase chain reaction–restriction fragment length polymorphism (PCR–RFLP) methods as previously described (16–18), with a standardised workflow as follows.

1. PCR amplification. For each variant, a genomic region flanking the polymorphic site was amplified using sequence-specific primers. PCR reactions were performed in a conventional thermocycler in 20–25 μL reaction volumes containing approximately 50–100 ng of genomic DNA, 0.2 μmol/L of each primer, 200 μmol/L of each dNTP, 1.5 mmol/L MgCl_2_ in 1× reaction buffer and 1 U of Taq DNA polymerase. A typical cycling protocol consisted of an initial denaturation at 95°C for 3 min, followed by 30–35 cycles of 95°C for 30 s, locus-specific annealing for 30 s and 72°C for 30 s, with a final extension at 72°C for 5 min.
2. Restriction digestion. PCR products were incubated with locus-specific restriction endonucleases that discriminated alleles by creating or abolishing cleavage sites. Digestion was conducted under the temperature and duration conditions recommended by the enzyme manufacturers, typically at 37°C for 2–4 h in the appropriate reaction buffer.
3. Gel electrophoresis and genotype calling. Digested fragments were separated by agarose gel electrophoresis alongside an appropriate DNA ladder. Banding patterns were visualised under UV illumination after nucleic-acid staining, and genotypes were assigned based on the expected fragment sizes corresponding to each allele.
4. Quality control. Negative (no-template) controls were included in each PCR batch to monitor contamination. A subset of samples was re-genotyped in duplicate to assess concordance. Samples with ambiguous banding patterns were re-amplified and re-digested. Overall genotyping call rates were recorded, and all genotype calls were performed blinded to case/control status.

### Hardy–Weinberg equilibrium and population checks

Hardy–Weinberg equilibrium (HWE) was tested in the control group for each locus prior to association analyses using chi-square tests. Variants showing substantial deviation from HWE (p < 0.001) were flagged for inspection, including re-checking of raw gels and repeat genotyping where necessary, and were further evaluated in sensitivity analyses to assess potential genotyping error or population stratification.

### Statistical analysis

All statistical analyses were performed using SPSS (version 26.0; IBM Corp., Armonk, NY, USA). Continuous variables were summarised as mean ± standard deviation (SD) or median (interquartile range) as appropriate, and categorical variables as counts and percentages. Group comparisons for continuous variables were conducted using Student’s t-test or non-parametric alternatives according to distributional assumptions; categorical variables were compared using chi-square tests or Fisher’s exact tests where expected cell counts were small.

For genetic association analyses, genotype and allele frequencies were compared between cases and controls. Logistic regression was used to estimate odds ratios (ORs) and 95% confidence intervals (CIs) for sepsis susceptibility, with adjustment for age and sex as primary covariates. Where appropriate, additive, dominant and recessive genetic models were evaluated, and model specifications were prespecified to reduce analytic flexibility.

Because multiple SNPs were analysed, we considered the potential inflation of type I error due to multiple testing. A conservative Bonferroni-corrected significance threshold (α_corrected = 0.05/number of loci tested) was used as a benchmark in sensitivity analyses, and associations that would remain significant under this threshold are explicitly highlighted in the Results. Unless otherwise specified, nominal two-sided p values < 0.05 were considered statistically significant.

Because detailed information on infection source/site, pathogen category and SOFA score was incomplete for some participants, these variables were not included in the primary genetic association models. Instead, they were examined descriptively and considered qualitatively when interpreting the cytokine and SNP associations, and their potential confounding effects are discussed as part of the study limitations.

## Results

### Clinical characteristics

Baseline demographic and clinical characteristics of the study population are summarised in **Table 1**. There were no significant differences between the sepsis and control groups with respect to age, sex distribution, body mass index (BMI), history of diabetes, history of hypertension, alcohol use or smoking (all p > 0.05). In the sepsis group, the mean temperature at diagnosis was 37.13 ± 1.15°C, the mean heart rate was 86.33 ± 8.42 beats/min, and mean systolic and diastolic blood pressures were 131.37 ± 21.56 mmHg and 85.32 ± 7.32 mmHg, respectively. Among patients with sepsis, the most frequent primary infection sites were abdominal and pulmonary sources, followed by limb, soft-tissue, catheter-related, head and face, and intracranial infections (**Table 1**).

**Table 1:** Comparison of baseline characteristics between the sepsis and control groups.

Parameters	Control group (n=840)	Sepsis group (n=480)	*p*
Gender Male Female	568 (67.6) 272 (32.4)	327 (68.1) 153 (31.9)	>0.05
age (X ± S)	62.12 ± 8.43	63.84 ± 9.55	>0.05
BMI (kg/m^2^, X ± S)	22.55 ± 2.04	23.57 ± 2.12	>0.05
DM history (%)	330 (39.3)	192 (40.0)	>0.05
Hypertension history (%)	341 (40.6)	203 (42.3)	>0.05
Drinking history (%)	262 (31.2)	144 (30.0)	>0.05
Smoking history (%)	336 (40.0)	200 (41.7)	>0.05
temperature (°C, X ± S)	/	37.13 ± 1.15	/
heart rate (time/min, X ± S)	/	86.33 ± 8.42	/
systolic pressure (mmHg, X ± S)	/	131.37 ± 21.56	/
diastolic pressure (mmHg, X ± S)	/	85.32 ± 7.32	/
infection site			/
abdominal infection	/	144 (30.0)	
pulmonary infection	/	119 (24.8)	
limb infection	/	56 (11.7)	
soft tissue infection	/	43 (9.0)	
catheter-related infection	/	40 (8.3)	
head and face infection	/	23 (4.8)	
intracranial infection	/	55 (11.5)	

### Serum Toll-like receptor and cytokine levels

**Figure 1:**
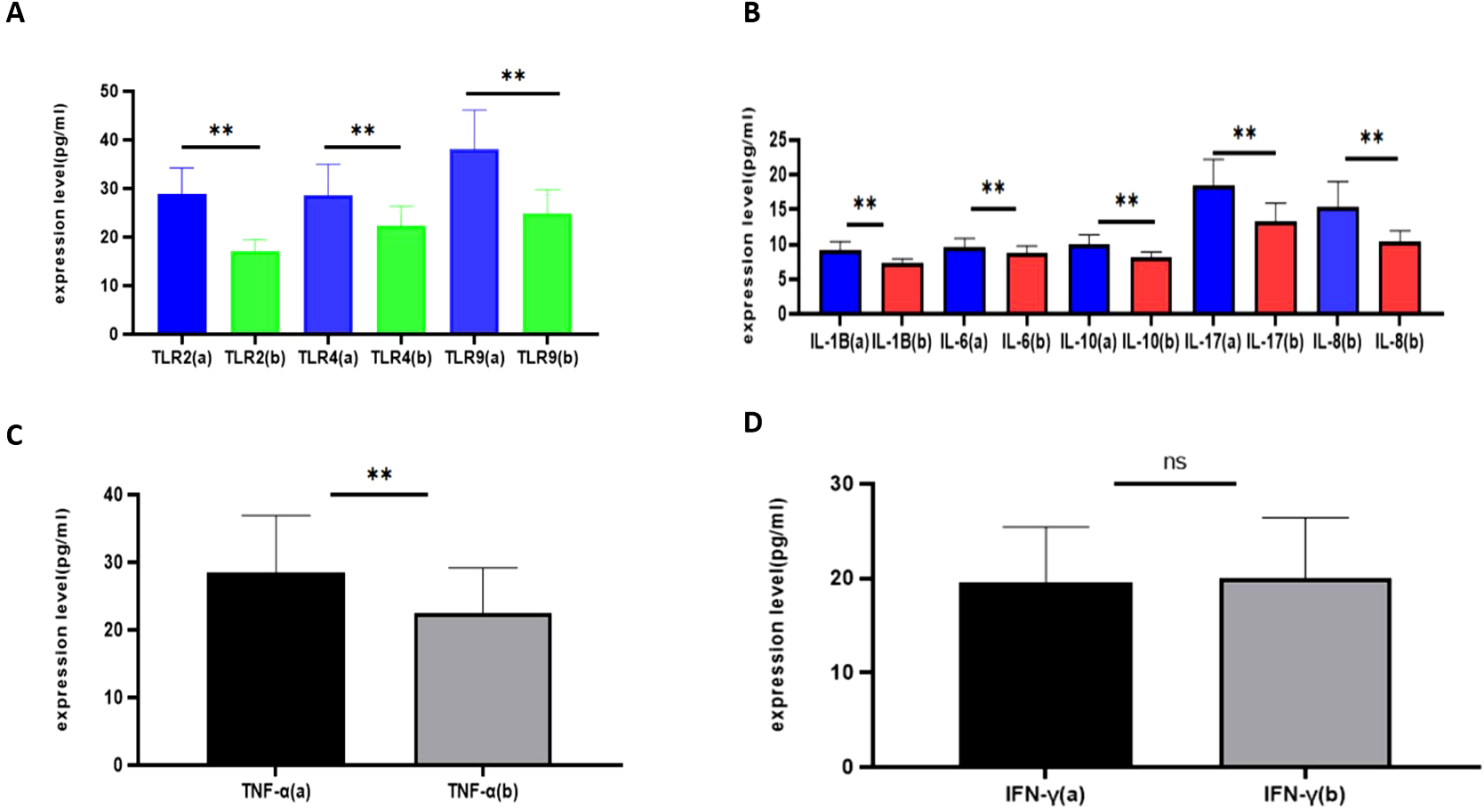
Serum Toll-like receptor and cytokine levels in patients with sepsis and healthy controls. **(A)** Serum TLR2, TLR4 and TLR9 levels. **(B)** Serum IL-1β, IL-6 and IL-8 levels. **(C)** Serum IL-10 and TNF-α levels. **(D)** Serum IFN-γ levels. Data are shown as mean ± SD. p < 0.05 versus controls. ** means p<0.05; ns means no significant difference.

Compared with healthy controls, patients with sepsis had significantly higher serum levels of TLR2, TLR4 and TLR9 (all p < 0.05). Likewise, the pro-inflammatory cytokines IL-1β, IL-6, IL-8 and TNF-α, as well as the anti-inflammatory cytokine IL-10, were markedly elevated in the sepsis group (all p < 0.05). In contrast, serum IFN-γ concentrations did not differ significantly between the two groups (p > 0.05). Overall, these findings indicate a pronounced TLR-driven pro-inflammatory milieu accompanied by a compensatory anti-inflammatory response, which is compatible with mechanisms proposed to contribute to neuroinflammation and blood–brain barrier dysfunction in sepsis, although central nervous system and BBB endpoints were not directly assessed in this study. The distributions of serum TLR and cytokine levels in the sepsis and control groups are shown in **Figure 1**.

### Gene polymorphism analyses

Genotype and allele frequencies of TLR2, TLR4 and TLR9 polymorphisms are presented in **Table 2**. For the TLR2 −196 to −174 del variant, the deletion (D) allele was more frequent in patients with sepsis than in controls, and both the I/D and D/D genotypes were associated with higher odds of sepsis compared with the I/I genotype in age- and sex-adjusted models, with the strongest effect observed for the D/D genotype. For TLR4, carriers of the A allele at rs1927911 (GA and AA genotypes) had a significantly increased risk of sepsis relative to the GG genotype. With respect to TLR9, the A allele of rs352140 (GA and AA genotypes) was positively associated with sepsis, whereas the C allele of rs574836 (TC and CC genotypes) was associated with lower odds of sepsis compared with the TT genotype (**Table 2**).

**Table 2:** TLR2, 4, 9 genotype and allele frequency for patients with sepsis.

TLR loci	Control group (N = 840)		Sepsis group (N = 480)		OR (95%CI) ^a^	P^a^
	n	Percentage (%)	n	Percentage (%)		
TLR2						
-196 to -174 del						
I/I	266	31.7	100	20.8	1.00^REF^	
I/D	258	30.7	145	30.2	1.49 (1.10-2.03)	**0.012**
D/D	316	37.6	235	49.0	1.98 (1.49-2.63)	**<0.001**
I	790	47.1	345	35.9	1.00^REF^	
D	890	52.9	615	64.1	1.58 (1.34-1.86)	**<0.001**
rs3804099						
TT	440	52.4	238	49.6	1.00^REF^	
TC	336	40.0	198	41.3	1.09 (0.86-1.38)	0.515
CC	64	7.6	44	9.1	1.27 (0.84-1.92)	0.305
T	1216	72.4	674	70.2	1.00^REF^	
C	464	27.6	286	29.8	1.11 (0.93-1.32)	0.252
rs3804100						
TT	456	54.3	270	56.3	1.00^REF^	
TC	326	38.8	170	35.4	0.88 (0.69-1.12)	0.326
CC	58	6.9	40	8.3	1.16 (0.76-1.79)	0.559
T	1238	73.7	710	74.0	1.00^REF^	
C	442	26.3	250	26.0	0.99 (0.82-1.18)	0.917
TLR4						
rs10759932						
TT	434	51.7	266	55.4	1.00^REF^	
TC	276	32.9	144	30.0	0.85 (0.66-1.10)	0.236
CC	130	15.4	70	14.6	0.88 (0.63-1.22)	0.490
T	1144	68.1	676	70.4	1.00^REF^	
C	536	31.9	284	29.6	0.90 (0.75-1.07)	0.232
rs1927911						
GG	370	46.4	114	23.8	1.00^REF^	
GA	354	42.2	244	50.8	**2.24 (1.71-2.92)**	**<0.001**
AA	116	11.4	122	25.4	**3.41 (2.45-4.75)**	**<0.001**
G	1094	67.5	472	49.2	1.00^REF^	
A	586	32.5	488	50.8	**1.93 (1.64-2.27)**	**<0.001**
rs11536889						
GG	485	57.7	298	62.1	1.00^REF^	
GC	332	39.5	162	33.8	0.79 (0.63-1.01)	0.064
CC	23	2.8	20	4.2	1.42 (0.76-2.62)	0.343
G	1302	77.5	758	79.0	1.00^REF^	
C	378	22.5	202	21.0	1.92 (0.76-1.11)	0.411
TLR9						
rs187084						
TT	260	31.0	143	29.8	1.00^REF^	
TC	336	40.0	229	47.7	1.24 (0.95-1.61)	0.127
CC	244	29.0	108	22.5	0.80 (0.59-1.09)	0.187
T	856	51.0	515	53.6	1.00^REF^	
C	824	49.0	445	46.4	0.90 (0.77-1.05)	0.196
rs352140						
GG	307	36.5	86	17.9	1.00^REF^	
GA	343	40.8	236	49.2	2.46 (1.84-3.39)	**<0.001**
AA	190	22.6	158	32.9	2.97 (2.16-4.08)	**<0.001**
G	957	57.0	408	42.5	1.00^REF^	
A	723	43.0	552	57.5	1.79 (1.53-2.10)	**<0.001**
rs574836						
TT	296	35.2	202	42.1	1.00^REF^	
TC	476	56.7	250	52.1	0.77 (0.61-0.97)	**0.034**
CC	68	8.1	28	5.8	0.60 (0.38-0.97)	**0.047**
T	1068	63.6	654	68.1	1.00^REF^	
C	612	36.4	306	31.9	0.82 (0.69-0.97)	**0.020**

OR, odds ratio; CI, confidence interval; ^a^Adjusted for age and sex in logistic regression models.

The bold values means significant difference.

Cytokine gene polymorphisms are summarised in **Table 3**. The T allele of IL-1B +3954 C/T and the TC and TT genotypes were strongly associated with increased sepsis risk compared with the CC genotype. The C allele of IL-6 −174 G/C showed a similar pattern, with GC and CC genotypes being more frequent in the sepsis group than in controls. For IL-10, the A allele of −1082 G/A (GA and AA genotypes) conferred higher odds of sepsis, whereas the C allele of −819 T/C and the CC genotype were modestly associated with lower odds of sepsis. No significant associations were observed for IL-8 or IL-17 polymorphisms in this cohort (**Table 3**).

**Table 3:** IL genotype and allele frequency for patients with sepsis.

IL loci	Control group (N = 840)		Sepsis group (N = 480)		OR (95%CI) ^a^	P^a^
	n	Percentage (%)	n	Percentage (%)		
IL-1B						
+3954 C/T						
CC	234	24.3	32	6.7	1.00^REF^	
TC	203	25.4	132	27.5	**4.75 (3.09-7.31)**	**<0.001**
TT	403	50.3	316	65.8	**5.73 (3.85-8.53)**	**<0.001**
C	671	37.0	196	20.4	1.00^REF^	
T	1009	63.0	764	79.6	**2.59 (2.15-3.12)**	**<0.001**
-511 C/T						
CC	434	51.7	240	50.0	1.00^REF^	
TC	337	40.1	197	41.0	1.06 (0.83-1.34)	0.689
TT	69	8.2	43	9.0	1.13 (0.75-1.70)	0.644
C	1205	71.7	677	70.5	1.00^REF^	
T	475	28.3	283	29.5	1.06 (0.89-1.26)	0.540
-31 C/T						
CC	444	52.9	262	54.6	1.00^REF^	
TC	335	39.9	175	36.5	1.16 (0.91-1.49)	0.253
TT	61	7.2	43	8.9	1.19 (0.79-1.82)	0.469
C	1223	72.8	699	72.8	1.00^REF^	
T	457	27.2	261	27.2	1.00 (0.84-1.19)	0.970
IL-6						
-174 G/C						
GG	274	31.7	110	22.9	1.00^REF^	
GC	250	30.7	145	30.2	**1.44 (1.07-1.95)**	**0.020**
CC	316	37.6	225	46.9	**1.77 (1.34-2.35)**	**<0.001**
G	798	47.5	365	38.0	1.00^REF^	
C	882	52.5	595	62.0	1.47 (1.25-1.73)	**<0.001**
-1363 G/T						
GG	392	46.7	231	48.1	1.00^REF^	
GT	350	41.7	174	36.3	0.84 (0.66-1.08)	0.192
TT	98	11.6	75	15.6	1.30 (0.92-1.83)	0.158
G	1134	67.5	636	66.2	1.00^REF^	
T	546	32.5	324	33.8	1.06 (0.89-1.25)	0.540
-1363 C/G						
CC	486	57.8	292	60.8	1.00^REF^	
GC	330	39.3	170	35.4	0.86 (0.68-1.08)	0.221
GG	24	2.9	18	3.7	1.25 (0.67-2.34)	0.596
C	1302	77.5	754	79.6	1.00^REF^	
G	378	22.5	206	20.4	0.94 (0.78-1.14)	0.567
IL-8						
-251 A/T						
AA	214	25.5	121	25.2	1.00^REF^	
AT	501	59.6	275	57.3	0.97 (0.74-1.27)	0.881
TT	125	14.9	84	17.5	1.19 (0.83-1.70)	0.389
A	929	55.3	517	53.9	1.00^REF^	
T	751	44.7	443	46.1	1.06 (0.90-1.24)	0.499
rs2227306						
CC	234	27.8	141	29.4	1.00^REF^	
CT	481	57.3	255	53.1	0.88 (0.68-1.14)	0.365
TT	125	14.9	84	17.5	1.12 (0.79-1.58)	0.597
C	949	56.5	537	55.9	1.00^REF^	
T	731	43.5	423	44.1	1.02 (0.87-1.20)	0.815
rs1126647						
AA	264	31.4	171	35.6	1.00^REF^	
AT	451	53.7	225	46.9	0.97 (0.74-1.27)	0.881
TT	125	14.9	84	17.5	1.19 (0.83-1.70)	0.389
A	979	58.3	567	59.1	1.00^REF^	
T	701	41.7	393	40.9	1.06 (0.90-1.24)	0.499
IL-10						
-592 C/A						
CC	234	27.9	124	25.8	1.00^REF^	
CA	336	40.0	228	47.5	1.28 (0.97-1.69)	0.090
AA	270	32.1	128	26.7	0.89 (0.66-1.21)	0.520
C	804	47.9	476	49.6	1.00^REF^	
A	876	52.1	484	50.4	0.93 (0.80-1.09)	0.416
-1082 G/A						
GG	366	43.6	70	14.6	1.00^REF^	
GA	322	38.3	256	53.3	4.16 (3.07-5.63)	**<0.001**
AA	152	18.1	154	32.1	5.30 (3.77-7.44)	**<0.001**
G	1054	62.7	396	41.3	1.00^REF^	
A	626	37.3	564	58.7	2.40 (2.04-2.82)	**<0.001**
-819 T/C						
TT	456	54.3	282	58.8	1.00^REF^	
TC	275	32.7	151	31.4	0.89 (0.69-1.14)	0.380
CC	109	13.0	47	9.8	**0.70 (0.48-1.01)**	**0.070**
T	1187	70.6	715	74.5	1.00^REF^	
C	493	29.4	245	25.5	**0.83 (0.69-0.99)**	**0.039**
IL-17						
rs2275913						
GG	434	51.7	242	50.4	1.00^REF^	
GA	336	40.0	198	41.3	1.06 (0.83-1.34)	0.690
AA	70	8.3	40	8.3	1.02 (0.67-1.56)	0.994
G	1204	71.7	682	71.0	1.00^REF^	
A	476	28.3	278	29.0	1.03 (0.87-1.23)	0.766
rs763780						
TT	453	53.9	267	55.6	1.00^REF^	
TC	328	39.0	173	36.0	0.89 (0.71-1.14)	0.394
CC	59	7.1	40	8.3	1.15 (0.75-1.77)	0.597
T	1234	73.5	707	73.6	1.00^REF^	
C	446	26.5	253	26.4	0.99 (0.83-1.19)	0.950

OR, odds ratio; CI, confidential index; ^a^Adjusted for sex and age by logistic regression model.

The bold values means significant difference.

For IFN-γ (**Table 4**), the T allele of +874 A/T and the TT genotype were associated with substantially higher odds of sepsis compared with the AA genotype, whereas the +2108 A/G variant did not show a clear association with sepsis. Similarly, for TNF-α (**Table 5**), the A allele of −308 G/A and the GA and AA genotypes were linked to an increased risk of sepsis, while the −376 G/A and −863 C/A polymorphisms were not significantly related to sepsis susceptibility.

**Table 4:** IFN-γ genotype and allele frequency for patients with sepsis.

IFN-γ loci	Control group (N = 840)		Sepsis group (N = 480)		OR (95%CI) ^a^	P^a^
	n	Percentage (%)	n	Percentage (%)		
+874 A/T						
AA	450	53.6	217	45.2	1.00^REF^	
AT	336	40.0	190	39.6	1.17 (0.92-1.49)	0.216
TT	54	6.4	73	15.2	**2.80 (1.90-4.13)**	**<0.001**
A	1236	73.6	624	65.0	1.00^REF^	
T	444	26.4	336	35.0	**1.50 (1.26-1.78)**	**<0.001**
+2108 A/G						
AA	485	57.7	298	62.1	1.00^REF^	
AG	332	39.5	162	33.8	0.79 (0.63-1.01)	0.064
GG	23	2.8	20	4.2	1.42 (0.76-2.62)	0.343
A	1302	77.5	758	79.0	1.00^REF^	
G	378	22.5	202	21.0	1.92 (0.76-1.11)	0.411

OR, odds ratio; CI, confidential index; ^a^Adjusted for sex and age by logistic regression model.

The bold values means significant difference.

**Table 5:** TNF-α genotype and allele frequency for patients with sepsis.

TNF-α loci	Control group (N = 840)		Sepsis group (N = 480)		OR(95%CI) ^a^	P^a^
	n	Percentage (%)	n	Percentage (%)		
-308 G/A						
GG	370	46.4	114	23.8	1.00^REF^	
GA	354	42.2	244	50.8	**2.24(1.71-2.92)**	**<0.001**
AA	116	11.4	122	25.4	**3.41(2.45-4.75)**	**<0.001**
G	1094	67.5	472	49.2	1.00^REF^	
A	586	32.5	488	50.8	**1.93(1.64-2.27)**	**<0.001**
-376 G/A						
GG	434	51.7	266	55.4	1.00^REF^	
GA	276	32.9	144	30.0	0.85(0.66-1.10)	0.236
AA	130	15.4	70	14.6	0.88(0.63-1.22)	0.490
G	1144	68.1	676	70.4	1.00^REF^	
A	536	31.9	284	29.6	0.90(0.75-1.07)	0.232
-863 C/A						
CC	485	57.7	298	62.1	1.00^REF^	
CA	332	39.5	162	33.8	0.79(0.63-1.01)	0.064
AA	23	2.8	20	4.2	1.42(0.76-2.62)	0.343
C	1302	77.5	758	79.0	1.00^REF^	
A	378	22.5	202	21.0	1.92(0.76-1.11)	0.411

OR, odds ratio; CI, confidential index; ^a^Adjusted for sex and age by logistic regression model.

The bold values means significant difference.

Because multiple loci were analysed, we further examined which associations would remain statistically significant under a conservative Bonferroni correction. With 28 loci tested, the Bonferroni-adjusted significance threshold was approximately p < 0.002. Under this criterion, the associations involving the TLR2 −196 to −174 del variant (D/D genotype and D allele), TLR4 rs1927911 (GA/AA genotypes and A allele), TLR9 rs352140 (GA/AA genotypes and A allele), IL-1B +3954 C/T (TC/TT genotypes and T allele), IL-6 −174 G/C (CC genotype and C allele), IL-10 −1082 G/A (GA/AA genotypes and A allele), TNF-α −308 G/A (GA/AA genotypes and A allele) and IFN-γ +874 A/T (TT genotype and T allele) remained statistically significant. In contrast, nominal associations for variants such as TLR9 rs574836 and IL-10 −819 T/C did not meet this more stringent threshold and should therefore be interpreted with greater caution.

**Figure 2:**
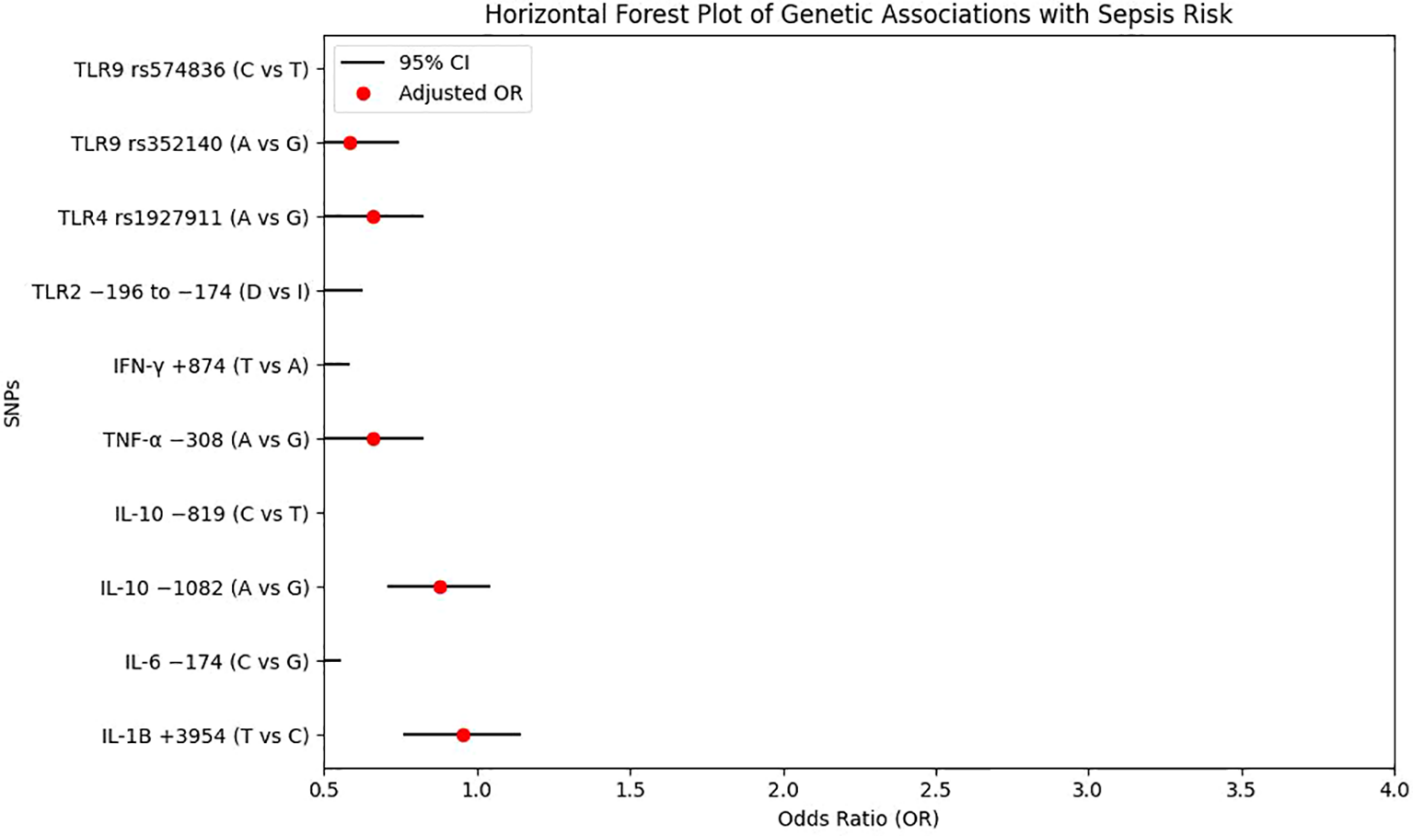
Forest plot of genetic associations with sepsis. Adjusted odds ratios (ORs) and 95% confidence intervals (CIs) for polymorphisms showing nominally significant associations with sepsis in age- and sex-adjusted logistic regression models.

To facilitate visual comparison of effect sizes across loci, a forest plot summarising the age- and sex-adjusted odds ratios and 95% confidence intervals for polymorphisms with nominally significant associations (p < 0.05) is provided in **Figure 2**.

## Discussion

Sepsis is a life-threatening organ dysfunction resulting from a dysregulated host response to infection and remains a major global health problem. Recent estimates suggest that sepsis affects tens of millions of individuals each year and accounts for a substantial proportion of global mortality across all age groups (19, 20). The host response in sepsis encompasses complex immune and non-immune alterations, including activation of innate and adaptive immune cells, endothelial injury, complement activation and coagulation disturbances (21–24). Rather than a simple switch from hyperinflammation to immunosuppression, most patients exhibit overlapping pro-inflammatory and anti-inflammatory responses, coupled with varying degrees of immune paralysis. These systemic processes can extend to the central nervous system, where excessive Toll-like receptor (TLR) signalling and cytokine release are thought to drive neuroinflammatory cascades and compromise blood–brain barrier (BBB) integrity, thereby contributing to sepsis-associated encephalopathy and long-term cognitive impairment. In the present study, however, central nervous system (CNS) involvement and BBB integrity were not directly measured, and their potential contribution is inferred from systemic TLR–cytokine profiles and existing mechanistic data.

In this hospital-based case–control study, patients with sepsis had significantly higher serum levels of TLR2, TLR4 and TLR9, together with elevated concentrations of IL-1β, IL-6, IL-8, IL-10 and TNF-α, compared with healthy controls. These observations are broadly consistent with previous reports describing marked increases in pro-inflammatory and counter-regulatory cytokines in sepsis (25–29). In contrast, IFN-γ levels did not differ significantly between groups, which aligns with studies suggesting that IFN-γ does not consistently increase in all septic populations and may even decline in the context of immune exhaustion or immunoparalysis (30, 31). Differences in study design, timing of blood sampling, analytical methods and patient characteristics likely contribute to discrepancies across the literature. Overall, our data reinforce the concept that sepsis is characterised by a TLR-driven inflammatory milieu. Activation of upstream receptors such as TLR2, TLR4 and TLR9 can engage MyD88-dependent pathways, leading to NF-κB activation and transcriptional up-regulation of IL-1β, IL-6, TNF-α and other cytokines. This in turn may promote endothelial activation, microvascular injury and disruption of barrier integrity, providing a plausible mechanistic link between systemic inflammation and the neuroinflammatory and BBB-related complications described in experimental and clinical studies.

Beyond serum profiles, we observed significant associations between sepsis risk and several polymorphisms in genes encoding TLRs and key cytokines. In age- and sex-adjusted models, variants in TLR2 (−196 to −174 del), TLR4 (rs1927911), TLR9 (rs352140 and rs574836), IL-1B (+3954 C/T), IL-6 (−174 G/C), IL-10 (−1082 G/A and −819 T/C), TNF-α (−308 G/A) and IFN-γ (+874 A/T) were related to sepsis risk. When a conservative Bonferroni correction was applied, a subset of these associations remained statistically significant, whereas others were attenuated, indicating that some findings are more robust than others and should be interpreted accordingly. Many of the implicated variants have been reported to influence promoter activity, mRNA stability or receptor structure, with downstream effects on transcriptional output and signalling capacity. It is therefore plausible that individuals carrying risk alleles have a heightened or prolonged TLR and cytokine response when exposed to pathogens, which may amplify systemic inflammation and aggravate organ dysfunction, whereas protective alleles may dampen excessive activation and confer partial resistance. Although our study was not designed to establish causality, the convergence between genetic associations and circulating cytokine patterns lends support to a genetic–inflammatory framework for sepsis pathogenesis.

At the mechanistic level, most of the identified variants map to components of canonical TLR–MyD88–NF-κB signalling pathways. TLR2 and TLR4 are prototypical pattern-recognition receptors that sense Gram-positive and Gram-negative components, respectively, and recruit adaptor molecules such as MyD88 to trigger downstream activation of NF-κB and AP-1, with subsequent transcription of IL-1β, IL-6, TNF-α and other mediators. TLR9 recognises unmethylated CpG motifs in microbial DNA and can also signal through MyD88-dependent cascades. Promoter polymorphisms in IL-1B, IL-6, IL-10, TNF-α and IFN-γ have been reported to modify cytokine production in response to inflammatory stimuli, shifting the balance between pro- and anti-inflammatory outputs. Taken together with our expression data, the present genetic associations are compatible with a model in which host polymorphisms tune the amplitude and duration of MyD88/NF-κB-dependent signalling in sepsis, thereby influencing systemic inflammation and, potentially, vulnerability to neuroinflammatory and BBB-related complications. Future functional studies will be required to dissect the precise molecular consequences of these variants in relevant cell types such as monocytes, endothelial cells and glia.

An important strength of this study is the relatively large sample size, including 480 patients with sepsis and 840 healthy controls from a Chinese population. This improves statistical power, reduces random error and allows more stable estimation of genotype and allele frequencies than many earlier studies that focused on single cytokines or small cohorts. China is characterised by marked diversity in ethnicity, climate and environmental exposures, which can shape both genetic background and infection patterns. Investigating TLR and cytokine polymorphisms in this context therefore provides valuable information on sepsis susceptibility in a large and heterogeneous population and adds to the limited data available from non-European cohorts. At the same time, the ethnic and regional specificity of our sample underscores the need for replication in other populations to assess generalisability.

The present findings also have potential clinical implications. First, the combination of serum TLR and cytokine levels with selected genetic markers may help identify patients at increased risk of sepsis or at higher likelihood of developing severe organ dysfunction, including sepsis-associated encephalopathy. Such information could contribute to risk stratification at the bedside and guide the intensity of monitoring and supportive care. Second, clarifying how specific polymorphisms modify TLR and cytokine signalling may inform the development and targeting of adjunctive therapies, for example, interventions that modulate TLR2/TLR4 signalling or downstream NF-κB activation in genetically susceptible individuals. Third, integrating these genetic and inflammatory markers into longitudinal studies that include neurological assessments, neuroimaging and BBB-related biomarkers may help to delineate how systemic signatures translate into sepsis-associated encephalopathy and longer-term cognitive outcomes, and to identify patients who might benefit most from neuroprotective strategies.

Several limitations should be acknowledged. First, although baseline demographic variables were comparable between the sepsis and control groups, we did not incorporate detailed clinical factors such as pathogen type, Sequential Organ Failure Assessment (SOFA) score or other severity indices into the genetic association models. These variables can influence cytokine expression and clinical outcomes and may act as potential confounders, and our odds-ratio estimates should therefore be viewed as minimally adjusted. Second, this was a single-centre study conducted in a specific ethnic population, which may limit the generalisability of the results to other settings and ethnic groups. Third, we focused on a panel of biologically plausible candidate polymorphisms. Although the number of tests was moderate and we applied conservative Bonferroni thresholds in sensitivity analyses, the possibility of residual false-positive findings due to multiple comparisons cannot be excluded, and independent replication is warranted. Finally, we did not directly measure neuroinflammation or BBB integrity; instead, their involvement is inferred from known TLR and cytokine biology and from systemic expression patterns. Future studies combining genetic data with cerebrospinal fluid biomarkers, advanced imaging and detailed neurological follow-up will be needed to validate these mechanistic links.

In conclusion, this study shows that sepsis in a Chinese cohort is characterised by a pronounced TLR-driven inflammatory profile and that specific polymorphisms in TLR2, TLR4, TLR9, IL-1B, IL-6, IL-10, TNF-α and IFN-γ are associated with sepsis risk, with several associations remaining robust after conservative correction for multiple testing. These combined expression and genetic data are compatible with enhanced TLR–MyD88–NF-κB signalling that may contribute to sepsis-related organ dysfunction and, potentially, neuroinflammatory and BBB-related complications. Our findings highlight the importance of considering both systemic inflammatory markers and host genetic background when investigating sepsis pathogenesis and support further work on biomarker-guided risk stratification and more personalised therapeutic strategies.

## Data Availability

The original contributions presented in the study are included inthe article/**Supplementary Material**, further inquiries can bedirected to the corresponding authors.
